# Nursing Her Wrath to Keep It Warm

**Published:** 1862-04

**Authors:** 


### "NURSING HER WRATH TO KEEP IT WARM."—*Burns.*

AND *she*—that is the heroine of our caption—should be employed as the Florence Nightingale of a whole regiment of wrath-nurses, if the "indifference" or "dislike" of the dental profession, here and in England, for each other, is to be nursed and kept warm through this entire generation, as hinted at by our Philadelphia correspondent. Don't mistake, reader. It is not intimated that it *ought* to be so perseveringly cherished. But it seems evident to us, if it exists now, which we doubt, that it will require even *wet-nursing* to keep it alive so long. Think of feeling indifferent to our professional brethren, or anybody else, through a whole generation. Pshaw! It can't be done. And then, to feel "indifference" or "dislike" without any cause! What if the conduct of the "rulers and the press of England" doesn't please us? The dentists neither administer the government nor edit the newspapers.

If we felt a dislike to some one we might possibly be persuaded to hold out (for appearance's sake) for a half hour or so;—but for a whole generation—" O. U. C." that is too long! W.

